# Synthetic low-density lipoprotein (sLDL) selectively delivers paclitaxel to tumor with low systemic toxicity

**DOI:** 10.18632/oncotarget.10493

**Published:** 2016-07-08

**Authors:** Hai-Tao Su, Xin Li, De-Sheng Liang, Xian-Rong Qi

**Affiliations:** ^1^ Beijing Key Laboratory of Molecular Pharmaceutics and New Drug Delivery System, School of Pharmaceutical Sciences, Peking University, Beijing, 100191, PR China; ^2^ State Key Laboratory of Natural and Biomimetic Drugs, Beijing, 100191, PR China

**Keywords:** synthetic low-density lipoprotein (sLDL), biomimetic, PTX-alpha linolenic acid (PALA), anti-tumor efficacy, low systemic toxicity

## Abstract

Low density lipoprotein (LDL), which is a principal carrier for the delivery of cholesterol, has been used as a great candidate for the delivery of drugs to tumor based on the great requirements for cholesterol of many cancer cells. Mimicking the structure and composition of LDL, we designed a synthetic low-density lipoprotein (sLDL) to encapsulate paclitaxel-alpha linolenic acid (PALA) for tumor therapy. The PALA loaded sLDL (PALA-sLDL) and PALA-loaded microemulsion (PALA-ME, without the binding domain for LDLR) displayed uniform sizes with high drug loading efficiency (> 90%). *In vitro* studies demonstrated PALA-sLDL exhibited enhanced cellular uptake capacity and better cytotoxicity to LDLR over-expressed U87 MG cells as compared to PALA-ME. The uptake mechanisms of PALA-sLDL were involved in a receptor mediated endocytosis and macropinocytosis. Furthermore, the *in vivo* biodistribution and tumor growth inhibition studies of PALA-sLDL were investigated in xenograft U87 MG tumor-bearing mice. The results showed that PALA-sLDL exhibited higher tumor accumulation than PALA-ME and superior tumor inhibition efficiency (72.1%) compared to Taxol^®^ (51.2%) and PALA-ME (58.8%) but with lower toxicity. These studies suggested that sLDL is potential to be used as a valuable carrier for the selective delivery of anticancer drugs to tumor with low systemic toxicity.

## INTRODUCTION

Cancer chemotherapy is a commonly accepted approach for the treatment of malignancies. However, most chemotherapeutic agents often cause severe side effects because they produce similar cytotoxicity in both cancerous and healthy cells. Therefore, the efficient and site-specific delivery of anticancer drugs to tumor has presented as a critical challenge for the success of cancer therapy.

Paclitaxel (PTX) is one of the most widely used compounds in the treatment of human malignancies but limited by its low water solubility (0.3 μg/mL) [1, 2]. Currently, Cremophor EL^®^ (polyoxyethylated castor oil) and ethanol at a ratio of 1:1 is used to increase the solubility of PTX and commercialized as Taxol^®^. However, Cremophor EL can cause many side effects like hypersensitivity reactions and neurotoxicity [3]. In order to avoid the adverse impacts of conventional formulations of PTX, many nano-preparations have emerged and culminated in the commercial product of Abraxane^®^, which permit higher doses of PTX over Taxol^®^, owing to an absence of Cremophor EL [4]. However, the expensive price and the severe toxicity caused through metabolism seriously limits the use of Abraxane^®^ [5]. So, there is a strong demand for the development of a more efficient and safer delivery system for PTX.

There is ample evidence that many types of cancer cells have unusually great cholesterol requirements. Low density lipoprotein (LDL) is a normal constituent in blood and acts as principal carrier for the delivery of cholesterol to tissues [6]. The recruitment of LDL to cancer cells makes LDL a great candidate for the delivery of different kinds of drugs and contrast agents by depositing in its lipophilic core or hydrophilic shell [7–9]. The main lipoprotein of LDL is apolipoprotein B-100 (apoB-100) which is a 550 kDa glycoprotein with nine amino acids (3359–3367, RLTRKRGLK) serving as the binding domain for LDL receptor (LDLR) [10, 11]. Glioblastoma is one of the most common and malignant primary brain tumors, which has a high mortality rate and short median prognosis due to its strong infiltration capacity and lots of angiogenesis [12, 13]. The LDLR is highly expressed at the blood brain barrier (BBB) and glioblastoma cells but sub-expressed in normal brain tissue cells [14–17], which makes LDL a potential drug carrier for brain tumor targeting delivery.

There are three ways to obtain LDL. One is extraction of LDL from mammalian serum, but it is difficult to isolate in large quantities, and the composition and size of isolated LDL is variable. Another approach is to use reconstituted LDL consisting of a lipid emulsion stabilized by purified apoB-100 [18, 19]. However, as a result of the large molecular weight and easy aggregation of apoB-100, it is not available to generate large quantities of reconstituted LDL by this approach. Recent studies have proved that it is possible to create a synthetic LDL (sLDL) using a lipid emulsion (ME) and a peptide composed of the LDLR binding domain of apoB-100 to substitute for serum LDL [11, 20, 21].

In this study, sLDL that mimics the LDL was chose as a carrier for the delivery of PTX to tumor. However, the loading efficiency of PTX into the sLDL is too low. Polyunsaturated fatty acids are important nutrients in the human body and play a great role on metabolism. Recent studies have showed that polyunsaturated fatty acids can reduce cardiovascular disease risk and exhibit anti-tumor activity through formation of bioactive lipid metabolites [22, 23]. Alpha linolenic acid (ALA) is one of the most-studied unsaturated fatty acids and has antiproliferation effect on tumor [24, 25]. We synthesized PTX-ALA conjugates (PALA) and hypothesized that PALA might improve the lipophilicity of PTX and subsequently enhance the loading efficiency of PTX in the sLDL. Then, the LDLR binding domain of apoB-100 was conjugated with DSPE-PEG and further modified onto the microemulsion surface to achieve the expected tumor targeting and therapy effect.

## RESULTS

### Synthesis of DSPE-PEG_2000_-Peptide and PALA

**Figure 1:**
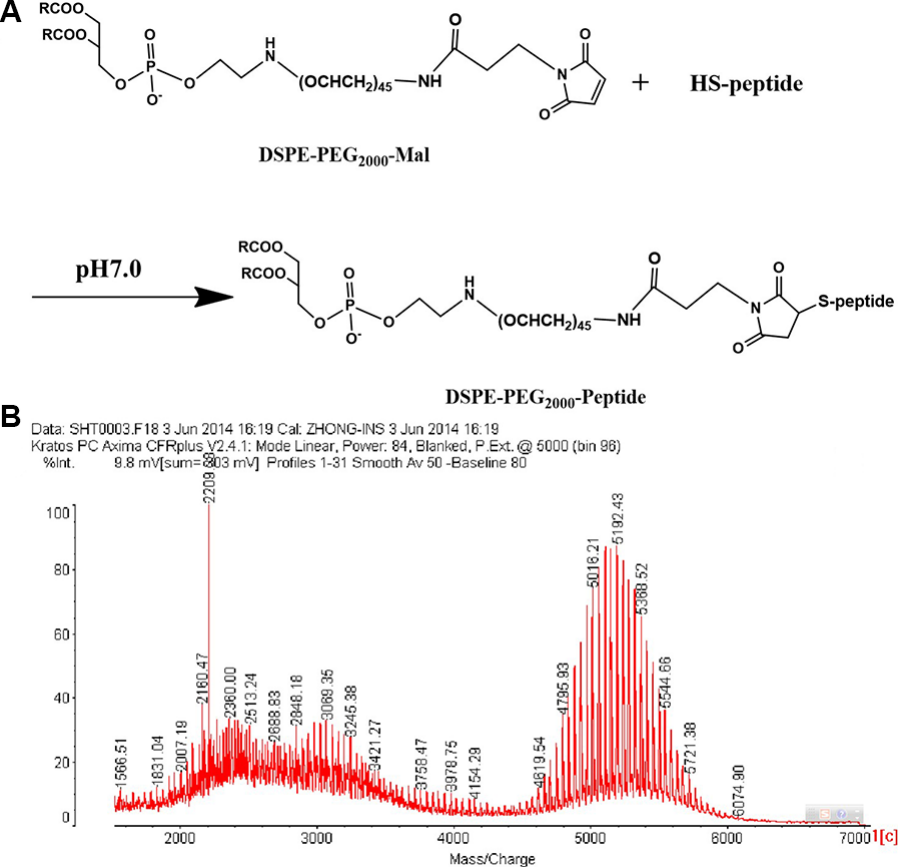
Synthesis schemes of DSPE-PEG2000-Peptide (A) and its MALDI-TOF MS (B)

DSPE-PEG_2000_-peptide (Figure 1A) was synthesized using DSPE-PEG_2000_-Mal and peptide by the Michael addition reaction. Successful synthesis was evidenced by the molecular shifts at about 5192.43 m/z, and each peak was spaced by 44 Da of the ethylene oxide monomer molecular weight in the MALDI-TOF MS analysis (Figure 1B). The molecular weight increment of DSPE-PEG_2000_-peptide from DSPE-PEG_2000_-MAL was about 2210 Da, which was in conformity with the molecular weight of the peptide.

**Figure 2:**
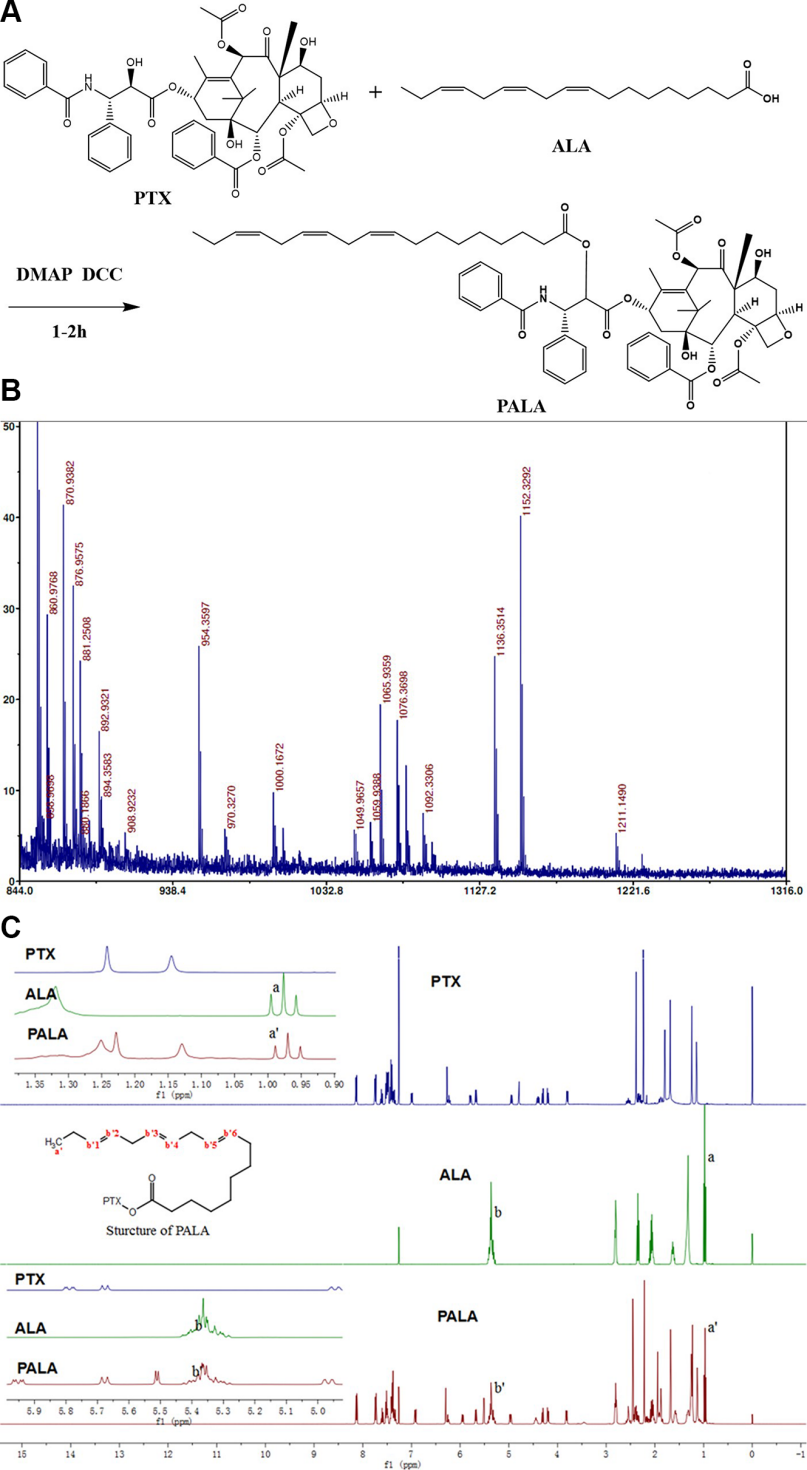
Synthesis schemes of PALA (A) and its MALDI-TOF MS (B) and ^1^H NMR (C)

PALA (Figure 2A) was synthesized from PTX and ALA in a single step by coupling ALA to PTX at the 2′-hydroxyl position. Successful synthesis was evidenced by the molecular shifts in the MALDI-TOF MS analysis (Figure 2B), in which the shifts at 1152, 1136 and 855 implied [PALA + K^+^], [PALA + Na^+^] and [PTX + H^+^], respectively. In addition, the identification of PALA was also confirmed by ^1^H NMR. Compared with PTX, the chemical shifts of PALA at 1 ppm and 5.35 ppm which respectively represented the hydrogen in the methyl and double bonds of ALA indicated the successful covalent linkage of PTX and ALA (Figure 2C).

### Preparation and characterization of PALA-ME and PALA-sLDL

**Figure 3:**
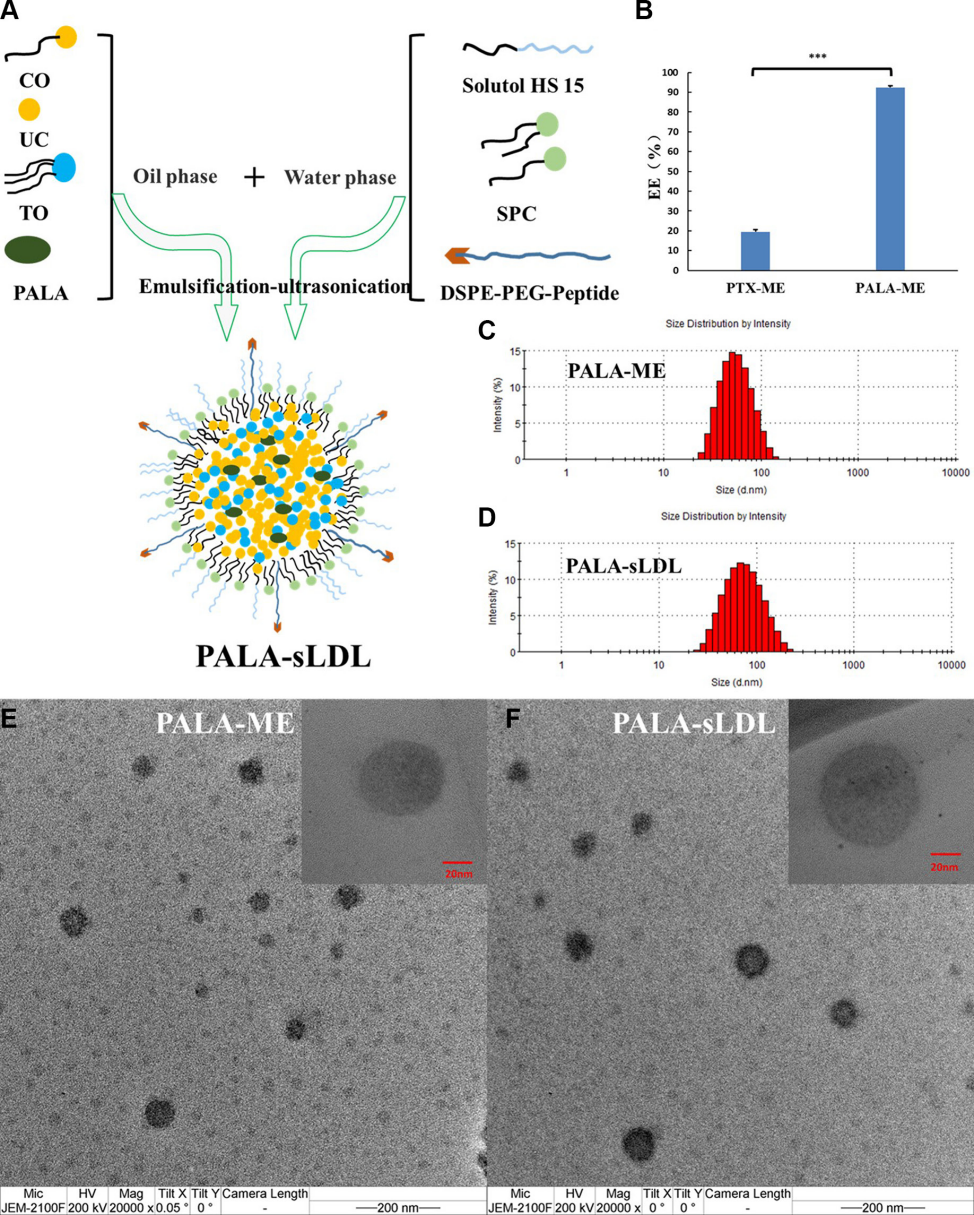
Preparation of PALA-sLDL (**A**). PALA-ME was prepared by an identical procedure but did not contain DSPE-PEG_2000_-peptide. The loading efficiency of PTX-ME and PALA-ME (*n* = 3) (**B**). Size distribution of PALA-ME (**C**) and PALA-sLDL (**D**) by dynamic light scattering. Transmission electron micrograph of PALA-ME (**E**) and PALA-sLDL (**F**).

PALA-ME and PALA-sLDL were prepared by a modified emulsification-ultrasonication method (Figure 3A). The difference between PALA-ME and PALA-sLDL was that PALA-sLDL contained the DSPE-PEG_2000_-Peptide while PALA-ME did not. The loading efficiency of PALA into PALA-ME was significantly higher than that of the PTX, which agreed with our anticipation (Figure 3B). As observed in Figure 3C–3E and 3F, both PALA-ME and PALA-sLDL displayed uniform sizes and spherical shapes. The mean particle size, size distribution and loading efficiency of PALA-ME and PALA-sLDL are shown in Table 1.

**Table 1:** Characteristics of nanoparticles (*n* = 3)

Sample name	Diameter* (nm)	Polydispersity index	Entrapment efficiency (%)
PALA-ME	51.80 ± 0.29	0.102 ± 0.017	92.36 ± 0.92
PALA-sLDL	66.80 ± 1.03	0.163 ± 0.015	95.39 ± 0.76

* the diameter was determined by dynamic light scattering.

### LDLR expression in U87 MG and HepG2 cells

**Figure 4:**
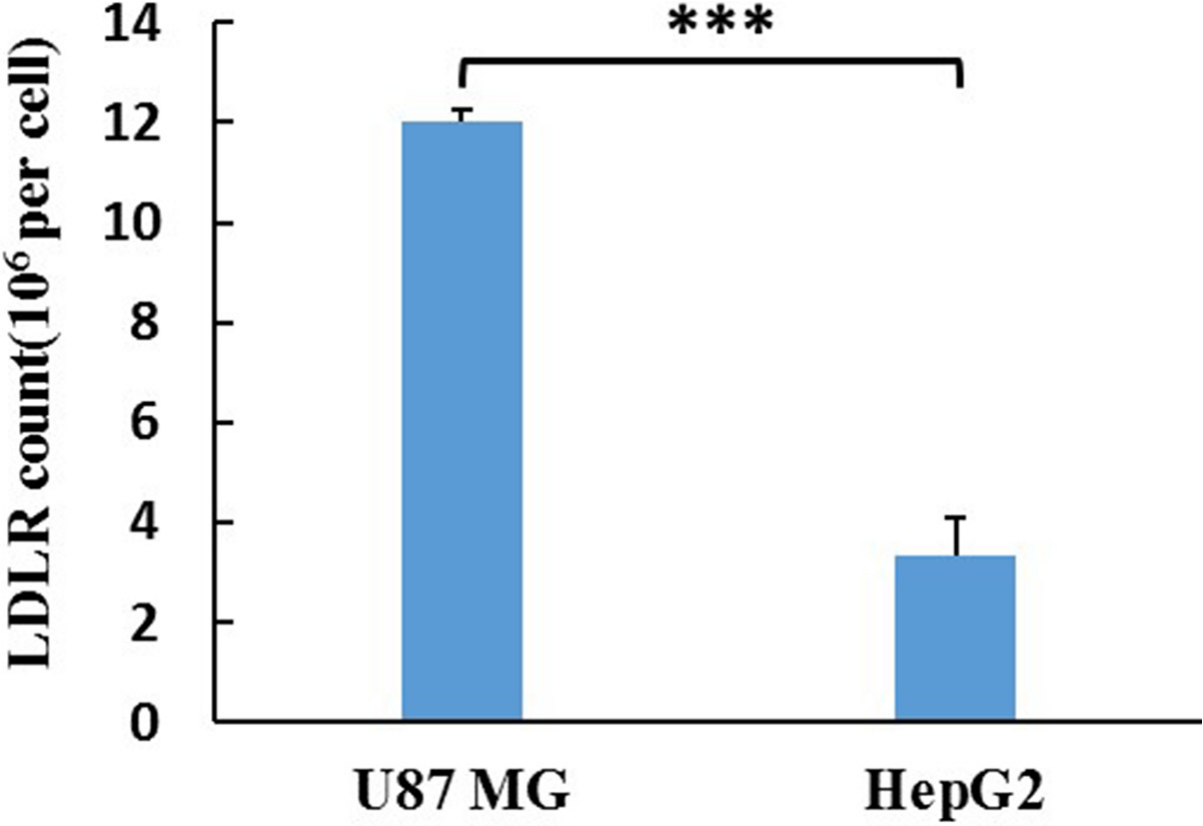
The LDLR count of a single cell The results are present as mean ± SD (*n* = 3).

ELISA was used for the detection of LDLR in U87 MG and HepG2 cells. The LDLR counts in U87 MG and HepG2 cells were assayed to be 22.96 and 6.38 ng/10^4^ cells. Considering the molecular weight of LDLR (115 kDa), the expression quantity of LDLR in U87 MG and HepG2 cell were calculated to be 1.20 × 10^7^ and 3.34 × 10^6^/cell (Figure 4), respectively.

### Cellular uptake in U87 MG and HepG2 cells

**Figure 5:**
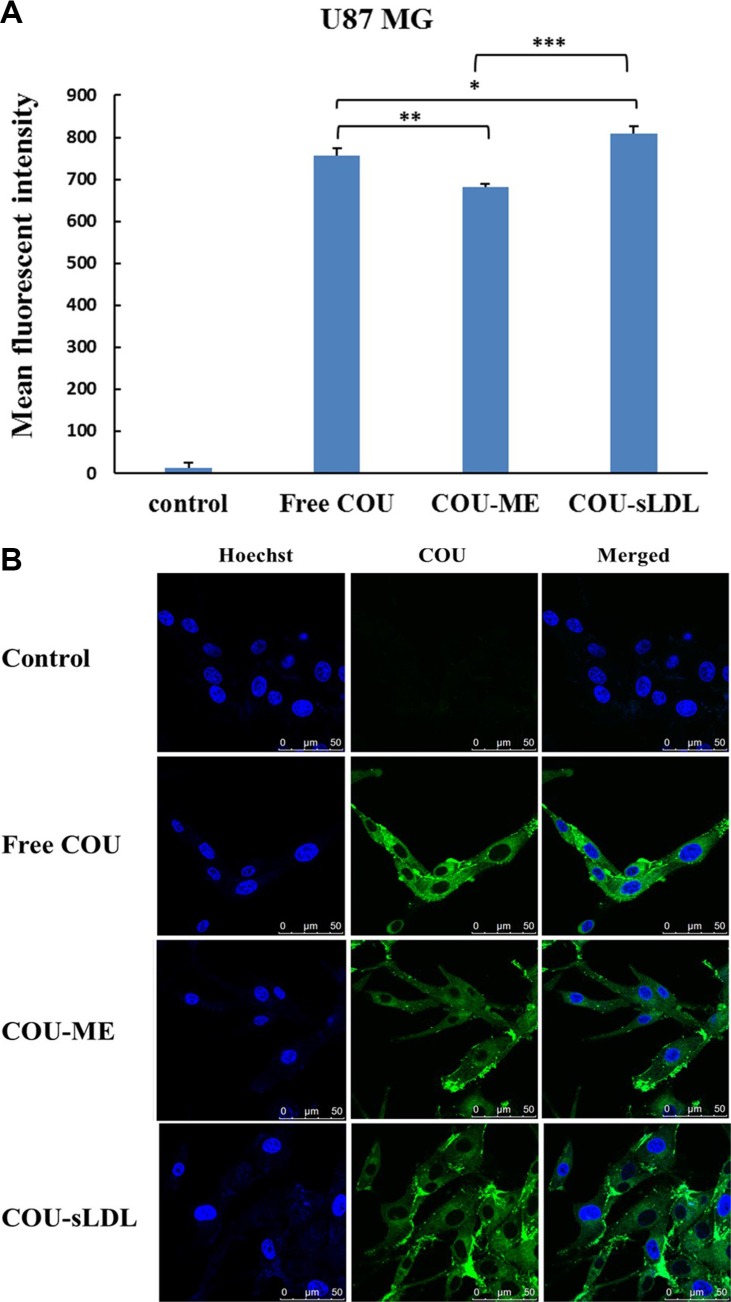
Uptake of COU by U87 MG cells determined by flow cytometric analysis (A) and by confocal (B) The results are present as mean ± SD (*n* = 3).

**Figure 6:**
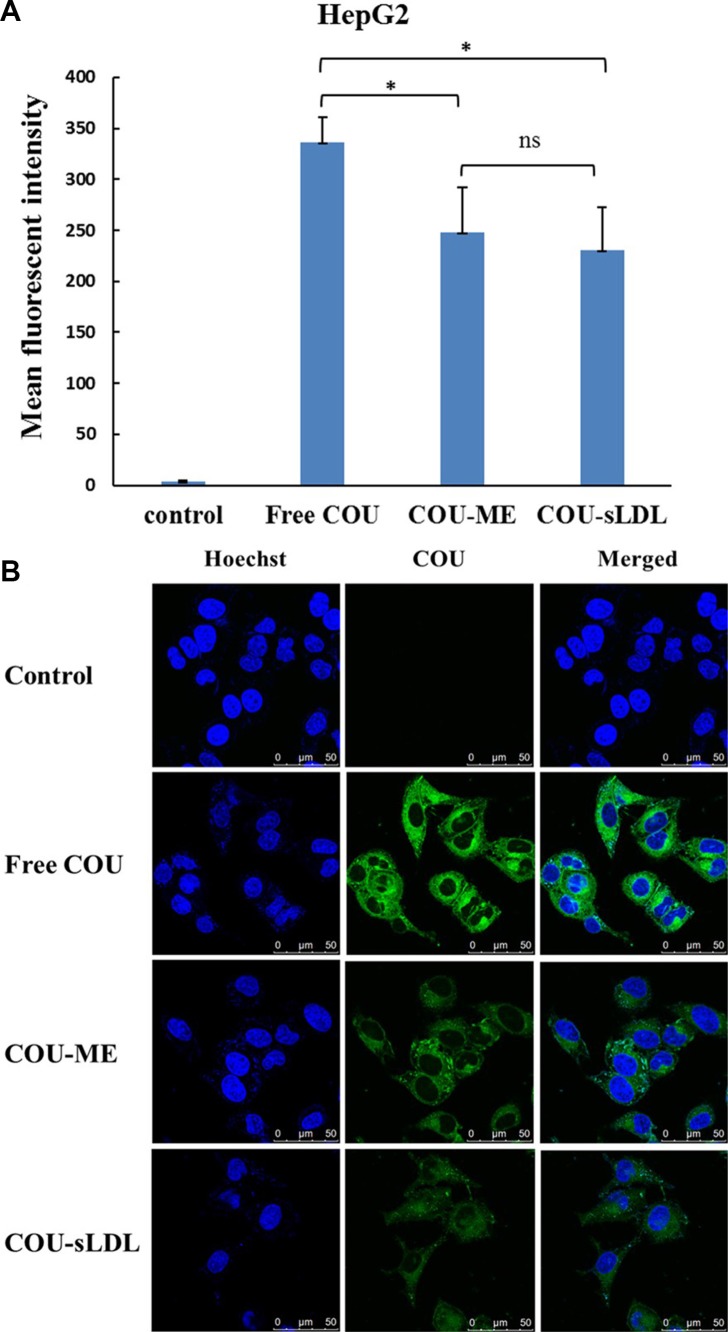
Uptake of COU by HepG2 cells determined by flow cytometric analysis (A) and by confocal (B) The results are present as mean ± SD (*n* = 3).

Cellular uptake was determined by the fluorescent of free coumarin-6 (COU) and COU loaded in different formulations (COU-ME and COU-sLDL) by flow cytometry and confocal laser scanning microscopy (CLSM) in U87 MG and HepG2 cells, respectively. After incubation for 2 h, significantly higher fluorescence intensity was observed in U87 MG cells after treated with COU-sLDL as compared to COU-ME, which indicated that the uptake of COU-sLDL was significantly increased due to the existence of sLDL (Figure 5A). Moreover, the same result was observed by a CLSM for U87 MG cells (Figure 5B). While for HepG2 cells, there was no significant difference in the fluorescence intensity of COU-sLDL and COU-ME groups (Figure 6A), which implied the uptake of COU-sLDL and COU-MR for HepG2 cells was similar. The result of CLSM for HepG2 cells (Figure 6B) was consistent with that acquired from flow cytometry.

### Cytotoxicity of PALA-sLDL

**Figure 7:**
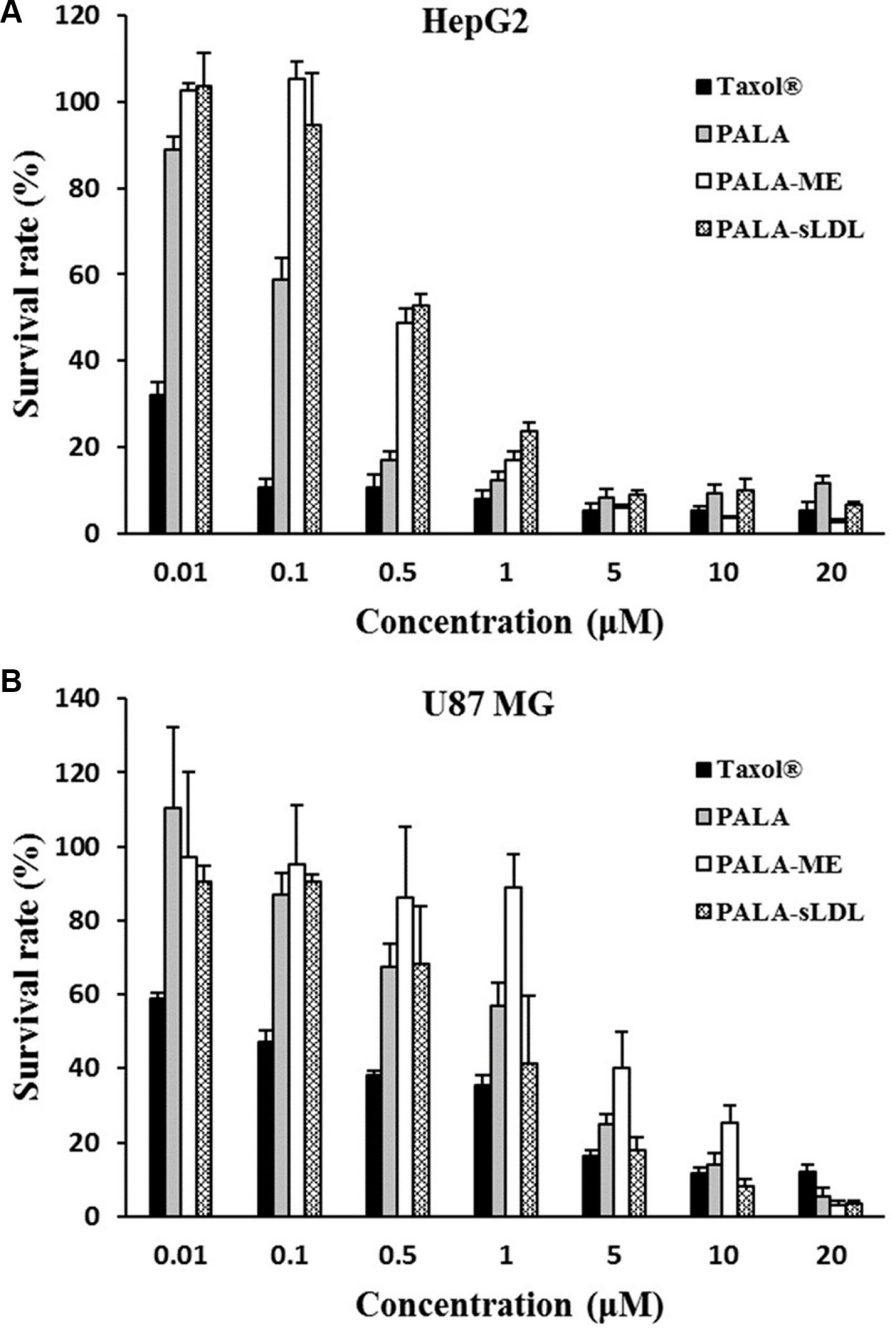
Cytotoxicity of different formulations on HepG2 (A) and U87 MG (B) The results are present as mean ± SD (*n* = 4).

CCK-8 was used to assay the cytotoxicity of Taxol^®^, PALA, PALA-ME and PALA-sLDL against HepG2 and U87 MG cells after incubation for 48 h. As shown in Figure 7, the viability of HepG2 and U87 MG cells were dependent on the PTX concentrations. At the low PTX concentration (< 1 μM), Taxol^®^ showed higher cytotoxicity than PALA, PALA-ME and PALA-sLDL. The cytotoxicity of PALA, PALA-ME and PALA-sLDL was increased with the PTX concentration increasing and almost to the same level when PTX concentration was more than 5 or 10 μM. The cytotoxicity of PALA-sLDL was weaker than that of PALA for HepG2 cells while the cytotoxicity of PALA-sLDL was stronger against U87 MG cells, which may be attributed to the different cellular uptake dose of PALA from different formulations. The IC_50_ of four formulations for different cell lines are shown in Table 2.

**Table 2:** IC_50_ (μM) of HepG2 and U87 MG cells treatment by different formulations (*n* = 4)

Cell lines	Taxol^®^	PALA	PALA-ME	PALA-sLDL
HepG2	0.0131 ± 0.0059	0.131 ± 0.023	0.319 ± 0.042	0.424 ± 0.111
U87 MG	1.14 ± 0.29	1.67 ± 0.28	4.98 ± 0.64	1.28 ± 0.17

### The receptor block experiment and the uptake mechanism of U87 MG cells

**Figure 8:**
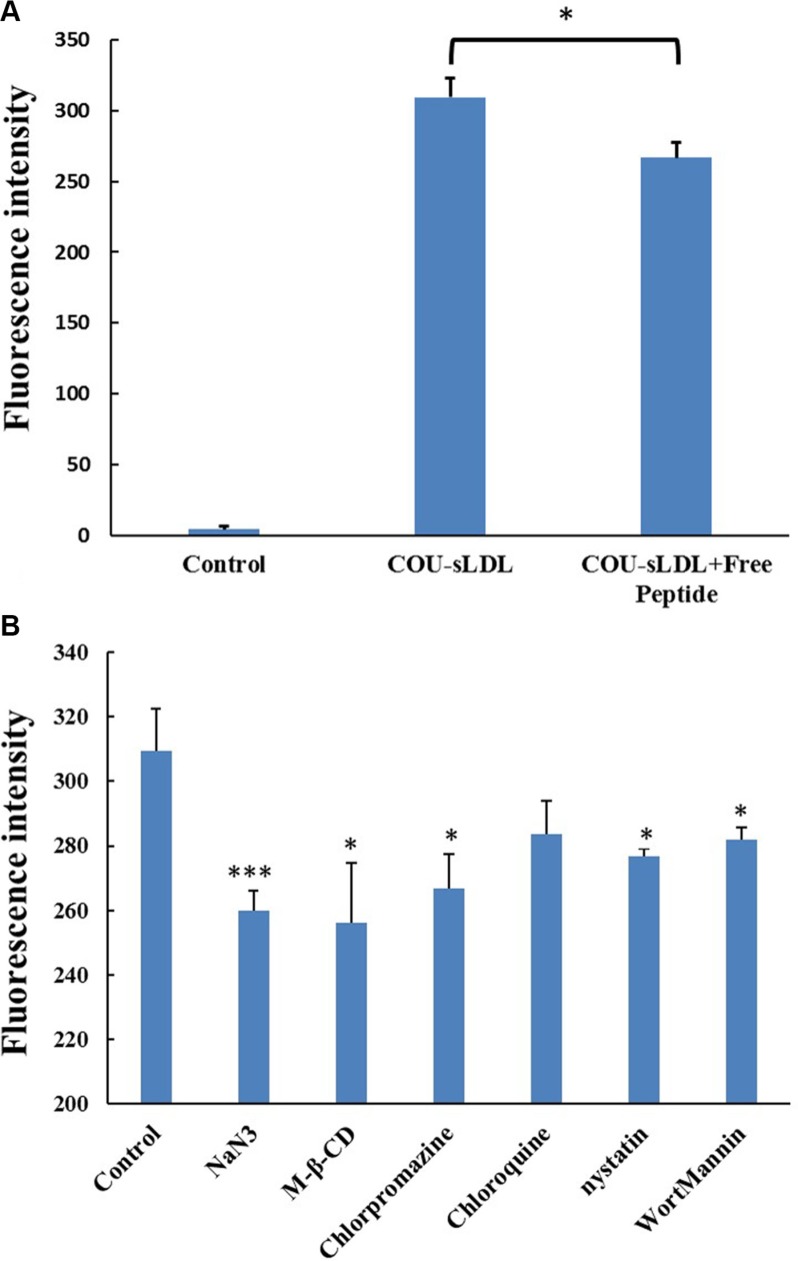
Receptor block experiment (A) and the uptake mechanism (B) of U87 MG cells The results are present as mean ± SD (*n* = 3).

In order to confirm the uptake mechanism of sLDL was related to the high-level expression of LDLR, we performed the competition block test by pre-incubation with excess free peptide to saturate the LDLR on the surface of U87 MG cells. Figure 8A showed that the fluorescence intensity of cells incubated with COU-sLDL was decreased after being blocked with free peptide for 0.5 h, indicating that the internalization of COU-sLDL was associated with the LDLR expression.

The effects of ATP depletion and different endocytosis inhibitors on the sLDL uptake were evaluated in U87 MG cells. As shown in Figure 8B, the uptake of COU-sLDL by U87 MG cells was significantly inhibited by NaN_3_ (ATP depletion agent), M-β-CD (cholesteroldepletion agent), chlorpromazine (clathrin-mediated endocytosis inhibitor), nystatin (caveolae-mediated endocytosis inhibitor) and wortmannin (macropinocytosis inhibitor), which indicated that the uptake of COU-sLDL was energy dependant and involved in caveolae--mediated endocytosis pathway, clathrin-mediated endocytosis pathway and macropinocytosis. Chlorpromazine (endosomal acidification inhibitor) did not inhibit the uptake of COU-sLDL, which implied that the uptake of COU-sLDL may be not related to endosomal acidification.

### Biodistribution *in vivo*

**Figure 9:**
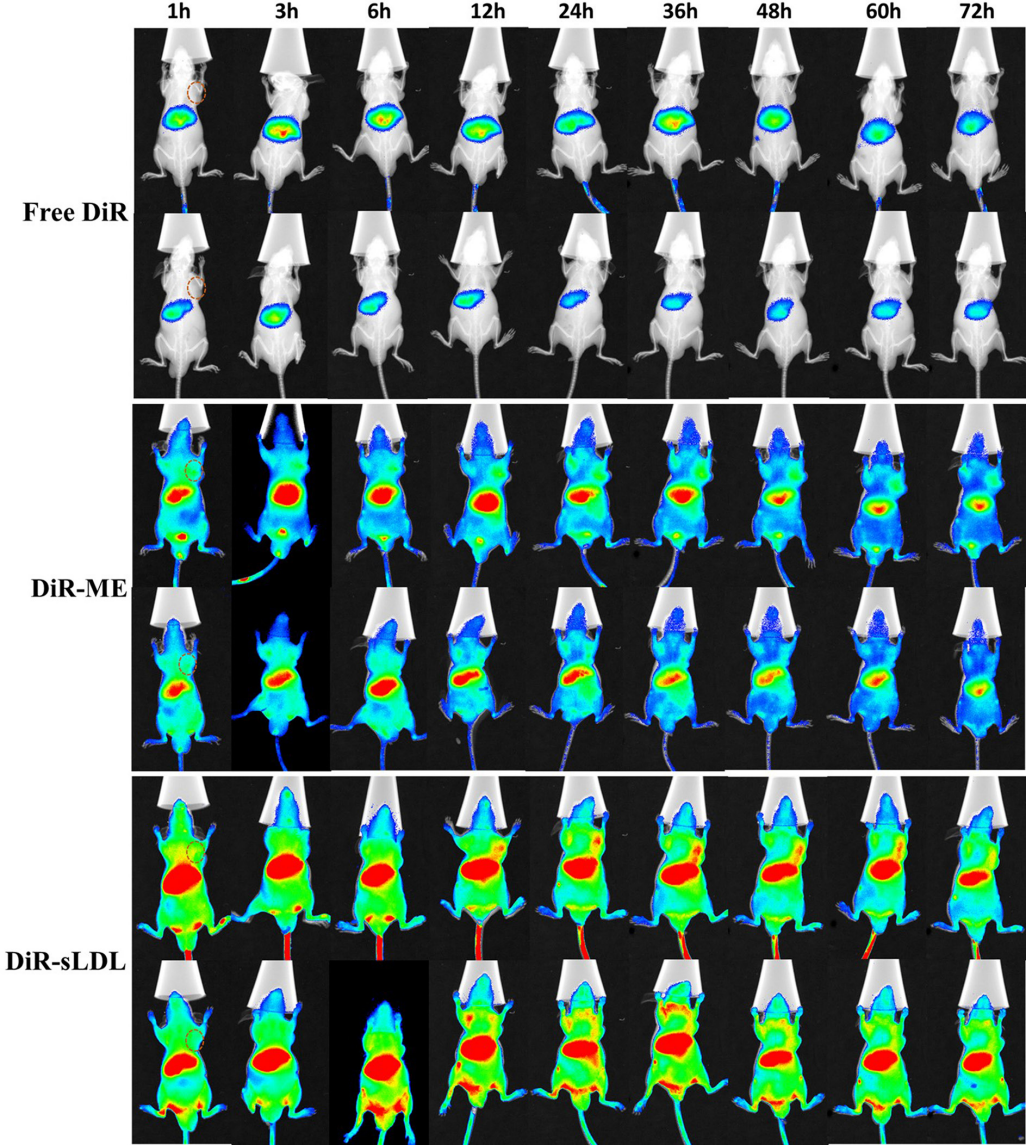
*In vivo* NIR fluorescence imaging of U87 MG tumor-bearing mice at 1, 3, 6, 12, 24, 36, 48, 60 and 72 h after iv injection of free DiR, DiR-ME and DiR-sLDL Tumor site is marked by dashed circle.

U87 MG cells (5.0 × 10^6^) were implanted subcutaneously in the right armpit of nude mice to establish a xenograft tumor model. Real-time fluorescence imaging was used to monitor the biodistribution of free DiR, DiR-ME and DiR-sLDL after administration until 72 h. As shown in Figure 9, free DiR eliminated from body rapidly. There was almost no visible distribution of free DiR in tumor. But for DiR-ME and DiR-sLDL, the fluorescence signals in tumor were observed to be increased over time and reached the maximum at 36 h, maybe contributed to the EPR effect. Moreover, compared with DiR-ME, the fluorescence intensity of DiR-sLDL accumulation in tumor was shown to be stronger at every time points, which could be explained by the synergistic targeting of EPR effect and the receptor mediated endocytosis of sLDL.

### Tumor growth inhibition *in vivo*

**Figure 10:**
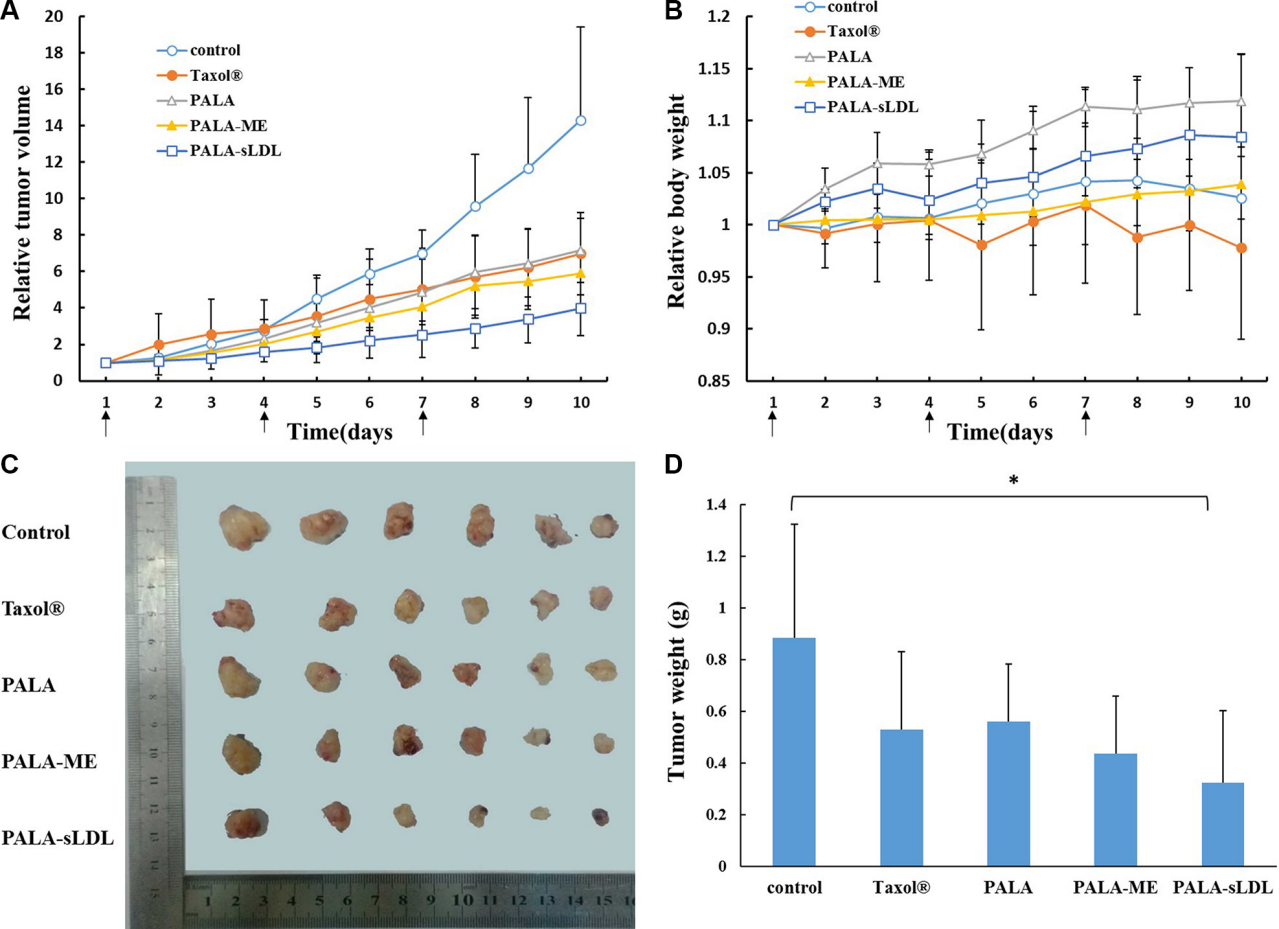
Tumor volume-time profile (A) and body weight-time profiles (B) of U87 MG tumor-bearing BALB/c nude mice after injected with 0.9% NaCl (control), Taxol^®^, PALA, PALA-ME and PALA-sLDL Photograph (**C**) and weight (**D**) of the solid tumors removed from different treatment groups at the study termination. The results are present as mean ± SD (*n* = 6).

Compared with the control, the tumor volume of mice after treated with Taxol^®^, PALA, PALA-ME and PALA-sLDL for 10 days (Figure 10A) was 2.1, 2.0, 2.4 and 3.6-fold decrease, respectively. The excised tumors from the mice treated with PALA-sLDL also exhibited the smallest size (Figure 10C) and weight (Figure 10D). These results indicated that PALA-sLDL produced the strongest anti-tumor effect *in vivo*. In addition, though the cytotoxicity of PALA was weaker than Taxol^®^ *in vitro*, PALA exhibited similar *in vivo* anti-tumor efficacy to Taxol^®^.

### Weight change of mice

Weight loss is an important indicator to monitor the adverse effects of tumor chemotherapy. The body weight (Figure 10B) of the mice treated with Taxol^®^ significantly decreased and was the lightest in all the groups, which indicated that Taxol^®^ had the strongest toxicity. In addition, the successively increased body weights of the mice respectively treated with PALA, PALA-ME and PALA-sLDL indicated that the covalent modification and nano-formulations could decrease the toxicity of PTX. Besides, the most weight gain of the mice treatment with PALA may be due to the nutritional role of polyunsaturated fatty acids.

### Immunohistochemical analysis of xenografts

**Figure 11:**
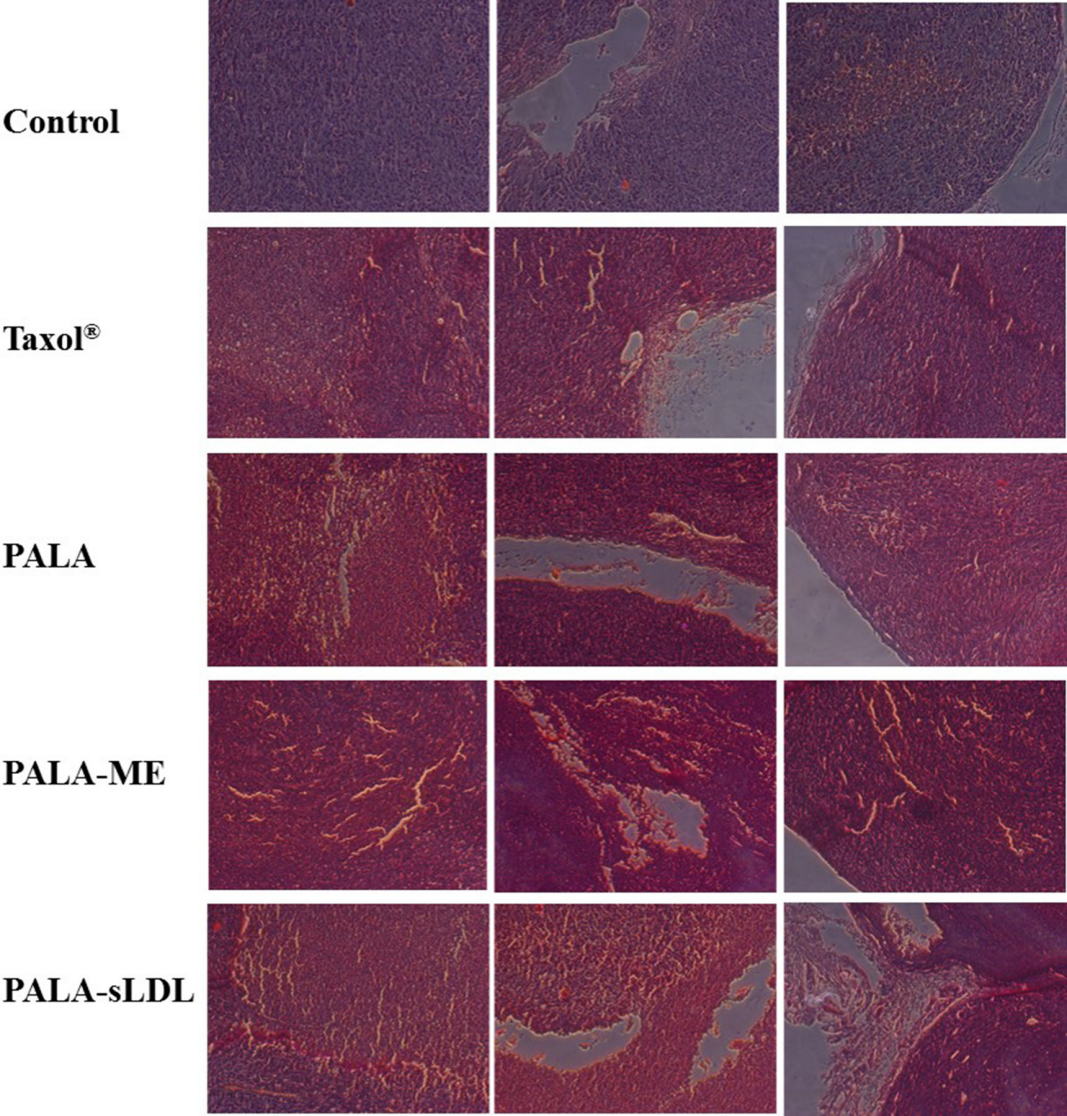
Histological (H&E) analysis of tumor samples from different treatment groups

The anti-tumor efficacy of PALA-sLDL was further confirmed by morphological and immunohistochemical analysis. Paraffin sections of the excised tumor were used for the H&E analysis. Compared to the control group, loose arrangement of cells, punctate or small focal area of necrosis, shrinkage and agglutination of nucleus and empty area were observed in all the groups of Taxol^®^, PALA, PALA-ME and PALA-sLDL (Figure 11), which indicated the changes in cell morphology and tumor tissue structure.

**Figure 12:**
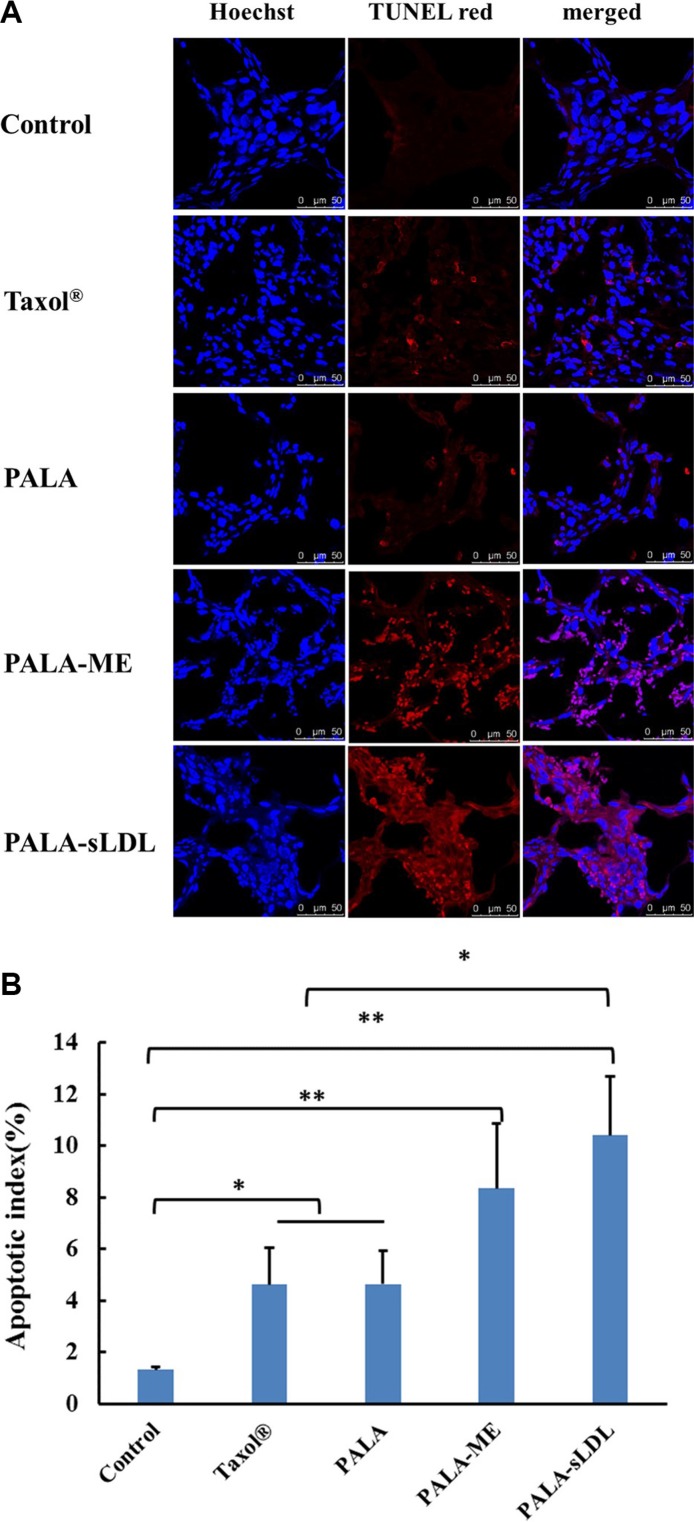
Therapeutic effect on apoptosis by TUNEL staining of the dissected tumor tissue (A) Apoptotic indexes of tumors in each group (**B**). The results are present as mean ± SD (*n* = 6).

TUNEL apoptosis detection was carried out using frozen sections of tumor tissues, in which the TUNEL red indicated the apoptosis of tumor cells (Figure 12A). All treatment groups exhibited positive TUNEL staining, and the apoptotic index in the groups of Taxol^®^, PALA, PALA- ME and PALA-sLDL were 3.50, 3.51, 6.31 and 7.88-fold increase over that of the control group (Figure 12B).

## DISCUSSION

LDL is a natural cholesterol carrier in vascular system. Nano-preparations that mimicked LDL have been studied in the delivery of drugs to tumors [20, 21]. A synthetic approach was adopted in this study to prepare the sLDL composed of the major composition of LDL and the LDLR binding domain (RLTRKRGLK) of apolipoprotein [10, 26]. PTX was initially selected to be encapsulated into the sLDL, but the loading efficiency was lower than 20% (as shown in Figure 3B). Given the strong hydrophobility of the core of LDL, it was speculated that increasing the lipophilicity of PTX could improve the loading efficiency of the drug. A study has demonstrated a 4-fold increase in incorporation of paclitaxel oleate to LDL compared to paclitaxel [27]. Thus, a fatty acid derivative, ALA, was selected to be conjugated to PTX to enhance the lipophilicity of PTX in our work. The ALA-PTX conjugation could increase the intermolecular interaction between drug and the core constituents of LDL, such as high lipophilic triglyceride and cholesteryl esters, that may be increase the loading efficiency of drug in the sLDL. It was inspiring that the loading efficiency of PALA was greatly increased to more than 90%. After loading of PALA, the diameter of PALA-sLDL was about 66 nm, which was considered to produce excellent extravasation and distribution in tumor tissues by EPR effect [28–31].

Though the *in vitro* cytotoxicity of PALA on HepG2 and U87 MG cells was weaker than PTX (Figure 7), the *in vivo* anti-tumor effect of PALA on U87 MG tumor-bearing BALB/c nude mice was similar to Taxol^®^ (Figure 10). More importantly, PALA exhibited a relatively low toxicity *in vivo* compared to PTX in terms of the body-weight change. The phenomenon can be attributed to the reason that the release of PTX from PALA is lower than Taxol^®^ since the process of decomposition of PALA was time-consuming, and the half-life of PALA is longer than PTX *in vivo* [32]. Moreover, the nutritional role of polyunsaturated fatty acids may resist the toxicity of PTX [33]. The similar results have been reported in the study of a squalenoyl-PTX, which exhibited weaker anti-tumor effect than PTX *in vitro*, while similar therapeutic result and milder body-weight changes and higher survival rate *in vivo* [34].

Liver is the main organ for the metabolism of LDL and LDLR is over-expressed in BBB [14, 15] and glioblastoma cells [16, 17]. HepG2 and U87 MG cell lines were selected to evaluate the anti-tumor activity of different preparations. However the cellular uptake and cytotoxicity of PALA-sLDL did not show significantly improvement than PALA-ME in HepG2 cells, but exhibited better effect on U87 MG cells (Figures 5, 6 and 7). These results was in corresponded with the level of LDLR expression in two kinds of cells (Figure 4), which indicated that the sLDL could be uptake by the cancer cells with higher expression of LDLR. Moreover, a study about levels of LDLR and LDLR-related protein shows that U87 MG cells had 2-fold greater LDL receptor and had 3.71-fold receptor-related protein content than HepG2 cells [35]. The mechanism of cellular uptake of LDL is receptor mediated endocytosis which has been proved by the receptor block experiment and the endocytosis inhibitors experiments (Figure 8).

*In vivo*, there was almost no distribution of free DiR in tumor, and the free DiR was eliminated rapidly and almost disappeared at 72 h (Figure 9). The accumulation of DiR-sLDL in tumor was higher than the DiR-ME and stronger fluorescence intensity could be detected until 72 h, indicating the excellent tumor accumulation capacity and long blood circulation half-life of DiR-sLDL. These characteristics are crucial factors for anti-cancer agents to produce eminent therapy effects and degressive systemic toxicity. In addition, liver and spleen is the main organs of reticuloendothelial system (RES), so the biodistribution of DiR-ME and DiR-sLDL in these tissues is higher than other part of the body.

For the U87 MG xenografted tumor-bearing mice, PALA-sLDL was expected to accumulate into tumor to enhance anti-tumor effect simultaneously via EPR effects and LDLR mediated targeting effect. As a result, the tumor inhibition efficiency of Taxol^®^, PALA, PALA-ME and PALA-sLDL was 51.2%, 50.0%, 58.8% and 72.1%, respectively, which demonstrated that PALA-sLDL had the strongest anti-tumor effect *in vivo* and its anti-tumor effect was higher than Taxol^®^ (Figure 10). Based on the results of morphological and immunohistochemical analysis, the strong anti-tumor effect of PALA-sLDL was demonstrated to be related to the enhancement of apoptosis of tumor cells and damages of tumor tissues. (Figures 11 and 12).

## MATERIALS AND METHODS

Distearoyl-glycerophosphoethanolamine-polyethyleneglycol_2000_-maleimide (DSPE-PEG_2000_-Mal) was purchased from NOF Corporation (Tokyo, Japan). Peptide (CYKLEGTTRLTRKRGLKLA) was custom synthesized by GL Biochem (Shanghai, China). Paclitaxel (PTX) was purchased from Norzer Pharmaceutical Co., Ltd. Alpha linolenic acid (ALA) was purchased from ANPEL Laboratory Technologies (Shanghai, China). Soybean phosphatidylcholine (SPC), unesterified cholesterol (UC), triolein (TO) and cholesteryl oleate (CO) were from Lipoid (Ludwigshafen, Germany), Wako Ltd. (Tokyo, Japan), Sinopharm Chemical Reagent Co., Ltd (Beijing, China) and InnoChem Science & Technology Co., Ltd. (Beijing, China), respectively. Solutol^®^ HS 15 was produced by BASF (Germany). Dicyclohexylcarbodiimide (DCC) and 4-dimethylaminopyridine (DMAP) were obtained from Sinopharm Group Co., Ltd. Cell Counting Kit*-8* (CCK- 8) was obtained from SolarbioScience & Technology Co., Ltd (Beijing, China). Coumarin-6 (COU) was purchased from Sigma-Aldrich Company (USA). TUNEL Kit was obtained from KeyGEN Biotech Co., Ltd. (Nanjing, China). Enzyme-linked Immunosorbent Assay (ELISA) Kit for LDLR was obtained from USCN Life Science Inc. Modified eagle medium (MEM), DMEM, penicillin-streptomycin, trypsin and Hoechst 33258 were obtained from Macgene Technology (Beijing, China).

### Cells culture

U87 MG cells (human glioblastoma cells) were cultured in MEM supplemented with 1% non-essential amino acids, 10% fetal bovine serum (FBS, GIBCO, USA), 100 IU/mL penicillin and 100 mg/mL streptomycin. HepG2 cells (human liver tumor cell) were cultured in DMEM supplemented with 1% non-essential amino acids, 10% FBS, 100 IU/mL penicillin and 100 mg/mL streptomycin.

### ELISA for the detection of LDLR

ELISA was used for the detection of LDLR in U87 MG and HepG2 cells. First of all, the U87 MG and HepG2 cells were broken by ultrasound, respectively. After ultracentrifugation at 10,000 rpm for 10 min, the supernatant was obtained. Then the diluted standard, blank and sample were operated following the manufacturer's protocol. Thereafter, the absorbance of each well was measured by an iMark microplate reader (Bio-Rad Laboratories, Hercules, CA, USA) at a wavelength of 450 nm.

### Synthesis of DSPE-PEG_2000_-Peptide

Peptide was conjugated with DSPE-PEG_2000_-MAL (1.2:1 molar ratio) in phosphate buffered saline (PBS, pH 7.4) at room temperature (20–25°C) for 6 h under vigorous stirring [36, 37]. The reaction mixture was dialyzed (molecular weight cutoff MWCO 3.5 kDa) in distilled water for 24 h to remove the unreacted peptides. The final solution was lyophilized and stored at −20°C until use. The obtained DSPE-PEG_2000_-peptide was confirmed by determining the molecular weight of the resulting DSPE-PEG_2000_-Peptide using MALDI-TOF MS.

### Synthesis of PTX-ALA conjugates (PALA)

PALA was synthesized from PTX and ALA in a single step that coupled ALA to PTX at the 2′-hydroxyl position [38]. To a solution of PTX (140 mg, 164 μmol) in methylene chloride (10 mL) under argon, DMAP (20 mg, 164 μmol), DCC (67.6 mg, 328 μmol), and ALA (46 mg, 164 μmol) were added. The reaction mixture was stirred at ambient temperature for 2 h. After dilution with appropriate diethyl ether, the reaction mixture was washed with 5% hydrochloric acid, water and saturated aqueous sodium chloride, sequentially. The mixture was dried in sodium sulfate and concentrated by rotary evaporator. Finally, radial chromatography (silica gel; ethyl acetate-hexane) was used to separate the product. ^1^H NMR and MALDI-TOF MS were used to characterize the product.

### Preparation of PALA-ME and PALA-sLDL

PALA loaded microemulsion (PALA-ME) was prepared by a modified emulsification-ultrasonication method [37, 39]. Briefly, PALA, triolein (TO), cholesteryl oleate (CO) and unesterified cholesterol (UC) were dissolved in an appropriate amount of ethanol and heated by a water bath to 60°C. Then, the ethanol was removed by heating and magnetic stirring for some min to form an oil phase. Solutol^®^ HS 15 and SPC were dissolved in PBS and kept in a water bath at 60°C to form an aqueous phase. The aqueous phase was added into oil phase under magnetic stirring at 600 rpm. Thereafter, the obtained primary emulsion was dispersed by ultrasound and put into ice bath until solidification dispersion was prepared. For the preparation of PALA loaded biomimetic low density lipoprotein microemulsion (PALA-sLDL), an identical procedure was followed except that DSPE-PEG_2000_-Peptide was used to replace an equivalent quantity of SPC. PALA-ME and PALA-sLDL was filtered through a 0.22 μm membrane filter to remove drug that was not loaded in nano-preparations.

### Characterization of PALA-ME and PALA-sLDL

The mean diameter and particle distribution of PALA-sLDL and PALA-ME were measured by dynamic light scattering (DLS) using a Malvern Zetasizer Nano ZS (Zetasizer 3000HS, Malvern, Worcestershire, UK) at 25°C. PALA-sLDL and PALA-ME were also morphologically characterized by a transmission electron microscope (TEM, JEOL, JEM-200CX, Japan).

The amount of PALA incorporated into PALA-sLDL and PALA-ME was determined by HPLC. The HPLC system consisted of LC-20AT Pump, SPD-20A UV detector and SIL-20A autosampler (Shimadzu, Japan). The mobile phase consisted of acetonitrile: double-distilled water [0–10 min (6:4), 10–12 min (6:4–0:10), 12–28 min (0:10), 28–30 min, (0:10–6:4)] introduced at a flow rate of 1 mL/min. The detection wavelength was 227 nm. An RP-18 column (4.6 mm × 250 mm, pore size 5 μm, Diamonsil^®^) was used. The loading efficiency was calculated from W_loaded drug_ / W_total drug_ × 100%, where W_total drug_ and W_loaded drug_ represents the amount of PALA before and after filtering by 0.22 μm membrane filter, respectively.

### Cellular uptake studies by flow cytometry and confocal laser scanning microscope

The U87 MG and HepG2 cellular uptake was investigated using flow cytometry by determining the fluorescent of different coumarin-6 (COU) loaded formulations. U87 MG and HepG2 cells were seeded in 6-well plates (Corning, NY, USA) at about 4 × 10^5^ cells/well and incubated for 24 h. Then, the medium in each well was replaced with fresh MEM or DMEM medium without FBS and containing free COU (100 ng/mL) or an equivalent concentration of COU loaded ME (COU-ME) and COU loaded sLDL (COU-sLDL) for 2 h under 5% CO_2_ at 37°C, respectively. After incubation, the cells were washed three times with cold PBS, detached with 0.05% trypsin and washed another three times with cold PBS to remove the COU that was not uptake by cells. Finally, the cells were resuspended in 0.5 mL PBS and detected by flow cytometry (Becton Dickinson, San Jose, CA, USA). The autofluorescence of the cells was used as a control.

Besides, a confocal laser scanning microscope (CLSM) was also used to confirm the cellular uptake with U87 MG and HepG2. Briefly, U87 MG and HepG2 cells were seeded in glass-bottom dishes at about 4 × 10^5^ cells/dish and incubated for 24 h. Then, the medium in each dish was replaced with fresh MEM or DMEM medium without FBS and containing free COU (100 ng/mL) or an equivalent concentration of COU-ME and COU-sLDL for 2 h under 5% CO_2_ at 37°C, respectively. After incubation, the cells were washed with cold PBS for three times and fixed with 4% formaldehyde for 10 min at 37°C. After another three rinses with cold PBS, Hoechst 33258 (5 μg/mL) was used to stain the cell nuclei for an additional 20 min at 37°C. A CLSM (Leica, Heidelberg, Germany) was used to image the cells. COU and Hoechst 33258 were excited using 488 nm and 345 nm lasers, respectively.

### Cytotoxicity assay

The cytotoxicity of Taxol^®^, PALA (6 mg/ml, dissolved in Cremophor EL: ethanol (1:1)), PALA-ME and PALA-sLDL against U87 MG and HepG2 cells was measured using the Cell Counting Kit*-8* (CCK-8). The cells were seeded into a 96-well plate at a density of approximate 5000 cells per well. After incubation for 24 h, the cells were treated with the various formulations at a range of concentrations for 48 h. Then, the medium in each well was replaced with 100 μl fresh cell medium containing 5% CCK-8 solution. After incubation for 2 h, the absorbance of each well was measured by an iMark microplate reader (Bio-Rad Laboratories, Hercules, CA, USA) at a wavelength of 570 nm.

### The receptor block experiment and the uptake mechanism evaluation

Flow cytometry described above was used for the LDLR receptor block experiment and the uptake mechanism evaluation. In the receptor block experiment, free peptide was added to the serum-free culture medium and incubated for 0.5 h before the addition of COU-sLDL. In the uptake mechanism experiment, NaN_3_ (1%), M-β-CD (10 mM), chloroquine (30 μM), chlorpromazine (20 μM), nystatin (25 μM) and wortmannin (0.8 μM) was respectively added to the serum-free culture medium and incubated for 0.5 h before the addition of COU-sLDL, too.

### *In vivo* biodistribution and tumor accumulation characteristics of sLDL

Male BALB/c nude mice (17–20 g) were purchased from Vital River (Beijing, China), and all of the animals were kept in standard housing conditions with free access to standard food and water. All care and handling of animals were performed with the approval of the Institutional Animal Care and Use Committee at Peking University Health Science Center.

U87 MG cells (5.0 × 10^6^) were implanted subcutaneously in the right armpit of nude mice. When the tumor volume reached approximately 400 mm^3^, the mice were randomly divided into 3 groups (*n* = 3 per group) and treated with free DiR, DiR-ME and DiR-sLDL by tail vein injection. The dose of DiR was 80 μg/kg. Then, after 1, 3, 6, 12, 24, 36, 48 and 72 h administration, the mice were anesthetized and visualized by a FX PRO Multi-mode *in vivo* imaging system (USA).

### *In vivo* tumor growth inhibition study

In order to evaluate the *in vivo* tumor inhibition efficacy, the tumor-bearing mice models were established according to the procedure described above. When the tumor volume reached approximately 100 mm^3^, the mice were randomly divided into 5 groups (*n* = 6 per group) and treated with 0.9% saline (as control group), Taxol^®^, PALA, PALA-ME and PALA-sLDL, respectively. The dose of PTX was 7.5 mg/kg. The tumor volume was calculated using the formula: Volume (mm^3^) = (*a* × *b*^2^)/2, where *a* and *b* are the major axis and minor axis of the tumor [37]. The tumor inhibition efficiency was evaluated by comparing the mean tumor volume of the treated mice (T) with that of the control mice (C), i.e. tumor inhibition efficiency = (1–T/C) × 100 (%). After 10 days, the mice were sacrificed and the tumor tissues were excised, weighed and photographed. The body weight of each mouse was monitored every day.

### Immunohistochemical analysis of xenografts

The pathological changes of tumor tissues that were removed from nude mice were evaluated using hematoxylin/eosin (H&E) staining. Briefly, tumors were excised at day 10, fixed in 4% paraformaldehyde solution, embedded by paraffin, and cut into 5-μm-thick sections. H&E staining was performed on all of the xenografts for analysis of morphology.

Apoptosis of tumor tissue was determined by the terminal deoxynucleotide transferase (TdT)-mediated dUTP nick-end labeling (TUNEL) assay. In brief, tumors were excised, frozen in OCT embedding medium and cut into 5-μm-thick sections. All frozen sections were detected by the *in situ* cell death detection kit (KeyGEN, Nanjing, China) following the manufacturer's protocol. The samples were analyzed using CLSM (Leica SP5, Heidelberg, Germany). The density of apoptotic cells was evaluated by the apoptotic index (AI%), which was defined as the percentage of apoptotic cells in total tumor cells.

### Statistical analysis

All data are presented as the means ± standard deviation (SD) of three or more samples. The student's *t*-test or one-way analyses of variance (ANOVA) was performed in statistical evaluation. A *P*-value of less than 0.05 was considered to be statistically significant (*P < 0.05, **P < 0.01, ***P < 0.005).

## CONCLUSIONS

In summary, synthetic low density lipoprotein (sLDL) was designed according to the characteristics of tumor growth, thus it can achieve tumor targeting drug delivery. Synthesis of PALA effectively solves the problem of low loading efficiency of PTX and decreases the systemic toxicity, and is a new attempt on the research of chemotherapy drug. Moreover, PALA-sLDL demonstrates an appropriate size (about 66 nm) and high loading efficiency, which greatly meet the requirements of optimal nanoparticle for cancer therapy. Furthermore, PALA-sLDL shows superiority in anti-tumor efficacy and lower toxicity against mice with U87 MG tumors compared to Taxol^®^. These result indicated that sLDL has great potential to be used as novel carriers to deliver hydrophobic chemotherapeutic drugs on tumor therapy.
